# The use of drama and theatre in enhancing communication skills of psychiatry trainees: a pilot study

**DOI:** 10.1192/bjo.2021.441

**Published:** 2021-06-18

**Authors:** Laura Stevenson, Farooq Khan, Qusai Bharmal, Opeyemi Odejimi, Sheliza Samnani, Polly Wright

**Affiliations:** 1 North Staffordshire Combined Healthcare NHS Trust; 2 Birmingham and Solihull Mental Health NHS Foundation Trust; 3 Black Country Healthcare NHS Foundation Trust; 4 The Hearth Centre

## Abstract

**Aims:**

Various methods have been employed in the development of communication skills. This pilot study was designed to assess the acceptability, feasibility and cost-effectiveness of a specially designed workshop exploring the use of drama and theatre in enhancing the self-reported communication skills of psychiatry trainees. As a secondary aim, it assessed if the value of the improvements translated both into clinical practice and to training situations, including success in the Royal College of Psychiatrists (RCPsych) Clinical Assessment of Skills and Competencies (CASC) examination.

**Method:**

A three-day drama and theatre workshop was organised in the West Midlands Deanery in conjunction with specialist instructors from performing arts at the Hearth Centre, Birmingham. The Tension State technique developed by Jacques Lecoq and Forum Theatre approach, were some of the methods employed to enable participants to develop the softer, but essential communication skills required for effective practice. Work was also undertaken focussing on self-regulation. Fourteen trainees completed the first day of the workshop. This pilot study utilised a mixed methodology to evaluate participants’ views of the perceived impact of using drama and theatre to enhance their communication skills. Feedback was obtained from organisers and facilitators specifically relating to feasibility and cost effectiveness. Data were collected from participants using pre and post-workshop questionnaires and focus groups.

**Result:**

All participants reported a positive and enjoyable experience, indicating that the approach was acceptable to those involved. The facilitators deemed this more novel approach to enhancing communication skills feasible, and cost effective and concluded that there was scope to incorporate it into routine psychiatry training in the area. It was however identified that the content of the workshop could be condensed, reducing the length therefore to two days. There was a notable increase in participants’ self-reported confidence in their communication skills post compared to pre-workshop. Trainees reported utilising the techniques in day-to-day practice. All of those participants who undertook the CASC examination during the workshop were successful, although it would be too presumptive to assume a causative effect. The workshop was completed without any adverse events and there were no concerns from a safety perspective.

**Conclusion:**

Drama and theatre, as a novel approach, appears to have noticeable benefits in enhancing the communication skills of psychiatry trainees. The success of this pilot study in demonstrating acceptability, feasibility and cost effectiveness, suggests that drama and theatre techniques could be easily incorporated into psychiatry training and potentially other medical education programmes.

